# Clinical practice of basin-shaped hepaticojejunostomy following hilar resection of stage III/IV hilar cholangiocarcinoma

**DOI:** 10.1186/s12876-019-1012-2

**Published:** 2019-06-20

**Authors:** Qi-jiong Li, Zhong-guo Zhou, Xiao-jun Lin, Xiang-ming Lao, Bo-kang Cui, Sheng-ping Li

**Affiliations:** Department of Hepatobiliary Pancreatic Oncology, Sun Yat-sen University Cancer Center, State Key Laboratory of Oncology in South China, 651 Dongfeng Road East, Guangzhou, 510060 Guangdong China

**Keywords:** Hilar cholangiocarcinoma (HCCA), Basin-shaped hepaticojejunostomy

## Abstract

**Background:**

Radical surgery for Bismuth type III/IV hilar cholangiocellular carcinoma, which was usually considered unresectable, seems to improve prognosis by increasing the surgical curability rate. However, the dilemma of multiple billiary stumps and high postoperative complication rate caused by hepato-enteric anastomosis has been the main impediment. Thus, we practiced and introduce a new technique called “basin-shaped” hepaticojejunostomy to improve the treatment.

**Methods:**

Thirty-two cases with Bismuth type III/IV hilar cholangiocarcinoma admitted to our department from Aug. 2013 to Dec. 2015 and who underwent hilar resection and resection segment 4(or plus resection segment 1) were reconstructed by “basin-shaped” hepaticojejunostomy. The clinical data were collected and analyzed.

**Results:**

All patients underwent successful R0 high hilar resection following basin-shaped hepaticojejunostomy and were discharged from the hospital without severe postoperative complications. The average operation time for hepato-enteric anastomosis was 42.1 ± 8.5 min. The postoperative bile leakage rate was 3.1% (1/32), and the biliary infection rate was 6.2% (2/32). Within a median follow-up of 25.6 months, none of the patients developed local recurrence around the hepato-enteric anastomosis.

**Conclusions:**

For patients with Bismuth type III/IV hilar cholangiocellular carcinoma who underwent resection segment 4(or plus resection segment 1), basin-shaped hepaticojejunostomy was a safe, simple and valid method for bile duct reconstruction, with a relatively low incidence of postoperative complications.

## Background

Hilar cholangiocarcinoma (HCCA) is the most common malignant tumor affecting the extrahepatic bile duct, including the total hepatic ducts and the right and left hepatic ducts, accounting for two-thirds of biliary tract tumors [1].

HCCA was first described by Klatskin in 1965, based on its distinctive clinical and pathological features [2]. The Bismuth-Corlette classification stratifies patients with HCCA into 4 types based on the extent of biliary involvement by the tumor [3]. Bismuth type IV tumors show extension into the secondary biliary radicals on both sides of the liver.

Resection of the involved intra- and extrahepatic bile ducts, as well as the associated hepatic lobe and caudate lobe, is the standard of care for patients with resectable tumors [4]. Over the past two decades, major hepatectomy or en-bloc resection with (extended) hemihepatectomy including the caudate lobe has been established as the mainstay of stage III and part of stage IV HCCA treatment if the range of invasion is mainly on one side of the primary bile duct. However, for the remaining stage IV HCCA patients with extended bilateral primary bile duct invasion, (extended) hemihepatectomy cannot achieve the curative criteria, while segment IV/extended IV is the best option to resect the tumor-invaded bile duct completely with R0 margins [5–7]. Although resection segment 4(or plus resection segment 1) could achieve a curative resection and abundant liver preservation, the very practical problem was that resection would leave multiple biliary stumps (usually more than 4, including secondary or tertiary biliary stumps).

In the conventional method, the multiple stumps are anastomosed one by one, or alternatively, the major branches are anastomosed, while the minor ones are ligated. These methods usually result in unsatisfactory anastomoses, leading to an elevated risk of complications and extended surgical time.

Non-synchronized with the improvement of resectability rates [8], the results remain unsatisfactory when followed by conventional methods for bilioenteric anastomosis, with high morbidity rates, ranging from 40 to 68%, and in-hospital mortality rates higher than those reported after liver resection for all other diseases [9]. The main cause of such high postoperative morbidity and mortality rates is septic complications [10], primarily due to reflux or leakage through the bilioenteric anastomosis following removal of the tumor. Since the main cause of postoperative complications is unsatisfactory bilioenteric anastomosis, the risk is even higher in patients with Bismuth types IV HCCA. Due to their extensive invasion range, Bismuth type IV HCCA patients always require resection segment 4 + 1 (including the caudate lobe) resection, which leaves multiple bile duct stumps to address. Thus, an improved method of biliary-enteric anastomosis for this situation is a pressing need.

Basin-shaped biliary-enteric anastomosis was first performed in 1987 to address iatrogenic bile duct injury and multiple bile duct stones followed by stenosis of the bile duct. The key procedure includes the following: 1) excision of the injured or constrictive bile duct; 2) incision and modification of bile stumps into one or several common openings to shape a basin with the jejunum opening for anastomosis; and 3) connection of the jejunum to the modified “basin shaped” opening and adjacent connective tissue of the Glisson pedicle by continuous sutures. The original intent was to take advantage of the wide-shaped anastomosis, avoiding multiple suturing of the iatrogenically caused (or constrictive) bile duct stumps one by one, which is almost impossible in many situations. Due to the improved techniques and science of HCCA surgery, more patients with stage IV HCCA have the opportunity for curative resection, resulting in more situations with multiple and adjacent stumps. Based on similar conditions, we practiced and improved the basin-shaped anastomosis method in HCCA resection. The biliary stumps were directly inserted into the basin-shaped anastomosis without modification.

In this study, we performed basin-shaped biliary-enteric anastomosis after resection segment 4(or plus resection segment 1) and high hilar resection for Bismuth type III/IV HCCA, and we observed and analyzed the safety and effects for these patients.

## Methods

### Patients

This study was approved by the ethics committee of Sun Yat-sen University Cancer Center and was performed in accordance with the Helsinki Declaration of 1975, as revised in 1983. From August 2013 to December 2015, consecutive patients with Bismuth III/IV HCCA without any satellite nodules or skip metastasis and distant metastatic spread who underwent curative resection underwent reconstruction with basin-shaped hepatico-enteric anastomosis were collected.

The diagnosis of HCCA was based on non-invasive criteria following the recommendations of the NCCN’s (National Comprehensive Cancer Network) Clinical Practice Guidelines in Oncology [https://www.nccn.org/] through radiology examination as enhanced CT (Computed X-ray Tomography) and MRCP (Magnetic Resonance CholangioPancreatography) preoperatively and was confirmed by postoperative pathological examination.

According to the Memorial Sloan-Kettering Cancer Center Classification [11], the criteria for unresectability were as follows: bilateral involvement up to the secondary biliary stumps; encasement of the main portal vein proximal to its bifurcation; atrophy of one hepatic lobe with encasement of the contralateral portal vein branch or with contralateral involvement of secondary biliary radicals; and distant metastases. The hepatectomy was classified by Brisbane classification [12].

### Preoperative preparation

(1) Admission examinations, including ultrasound, multi-phase CT, and MRCP, were performed in our institutions was to facilitate analysis of the tumor’s invasion of the left and right hepatic ducts and their relationships with the portal vein and hepatic artery. Data acquisition was based on hospital records and follow-up information, including age, sex, bilirubin levels, and alkaline phosphatase levels. (2) All of the patients were jaundiced on admission; patients whose TBil (Total Bilirubin) level was higher than 300 μmol/L underwent percutaneous transhepatic cholangial drainage before surgery. (3) Patients admitted with biliary tract infections were treated with antibiotics.

### Surgical procedure

Exploratory laparotomy was performed through an upper midline incision with right or bilateral extensions (an inverse “L”-shaped or Mercedes logo-shaped incision). Tumor resectability based on the exclusion of hepatic metastases, peritoneal implants, regional lymph node involvement, and vascular invasion, was carefully estimated with ultrasound guidance. Ultrasonography was also used in all cases to identify portal vein and hepatic artery involvement, as well as to assess adequate residual liver volume and ensure at least one portal vein and hepatic artery main branch could be separated from tumor and be reserved or could be restricted if involved by tumor.

After the exploration and determination of resectability, radical resection of hilar cholangiocarcinoma was performed in all of the enrolled patients. In order to preserve the artery supply of the remnant bile duct, we tried to protect the branches at the location of 3 and 9 o’clock of the cross section of bile ducts as far as possible. Subsequently, the distal extrahepatic bile duct was sutured closed, and the proximal bile duct was mobilized from the portal vein and hepatic artery under direct visualization in preparation for bilioenteric anastomosis. All biliary radicals were probed to exclude any stricture in the proximal parts of any biliary stump and to reestablish the continuity before hepaticojejunostomy.

**Fig. 1:**
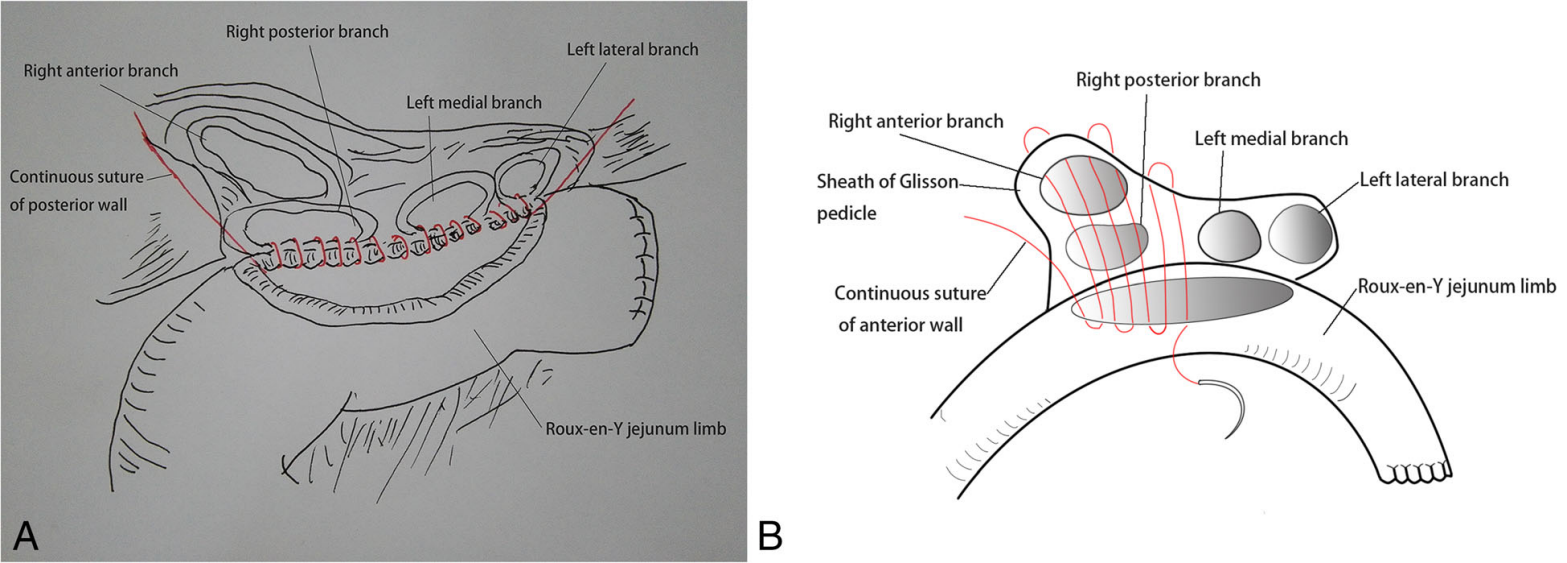
End-to-side anastomosis of the biliary sheath to the Roux-en-Y jejunum limb. The range of the “basin” was determined by the distance between the multiple bile ducts, which can only envelop the bile ducts. Therefore, the wall of the “basin” includes the outside part of the bile duct and the connective tissue and sheath of the Glisson pedicle. **a** Continuous single-layer sutures of the posterior wall of the anastomosis. Bile duct walls on the posterior wall and the sheath of the connective tissue and sheath of the Glisson pedicle were sutured with the jejunum limb. **b** Continuous single-layer sutures of the anterior wall of the anastomosis after completion of the posterior wall with another independent PDS or Proline stitch. The stitch was tied up to posterior one to complete the entire anastomosis

**Fig. 2:**
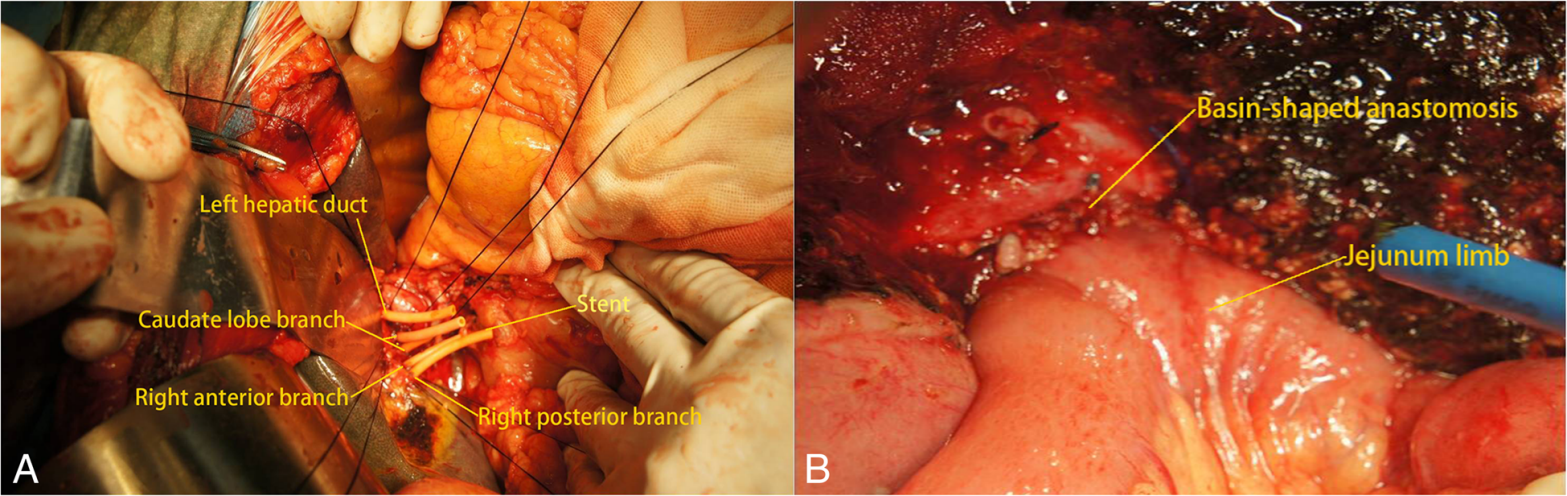
Intraoperative photos before and after anastomosis. **a** Before anastomosis, 5 bile duct radicals were lined up and stented with a latex tube. **b** After anastomosis, all 5 bile duct radicals were inserted and fixed to the jejunum limb. In our current clinical practice, we continue to make improvements based on our experience: (1) 4–0 PDS II or Proline stitches are recommended, while silk stitches are discouraged in an effort to avoid biliary calculus deposition. (2) Insertion of a stent is necessary for every bile duct radical to prevent postoperative stricture. (3) The diameter of the basin-shaped anastomosis should be only sufficiently large to allow every bile duct radical to be inserted; a larger size only causes more irritation to the remnant facet of the liver

The steps for basin-shaped hepaticojejunostomy were as follows: (1) skeletonize the proximal hepatic duct and the intrahepatic biliary tree, ensuring that every skeletonized bile duct is at least 0.5 cm in length for bilioenteric anastomosis; (2) transect the jejunum at 15 cm distal from the Treitz ligament, suture the distal end of the jejunum limb to close and bring it up in a retrocolic fashion; (3) attach the stump of the jejunum limb to the peritoneum, and incise the jejunum limb on the contralateral side against the mesentery more than 4 cm distal from the sutured stump of the jejunum limb; (4) use single-layer continuous sutures with 4–0 or 5–0 absorbable monofilament material to suture the posterior wall of the multiple bile stumps and the sheath with the connective tissue of the Glisson pedicle between the bile stumps as the posterior layer of the anastomosis (Fig. 1a); (5) insert a 5-French polyvinyl chloride tube into each biliary stump as a stent; all of the biliary stumps are drained into this “biliary basin”; (6) perform the same procedure as the posterior layer is to suture the anterior layer of the anastomosis (Fig. 1b); and (7) accomplish end-to-side anastomosis of the proximal jejunum limb to the 30–40 cm distal part from the biliary enteric anastomosis site on the distal jejunum limb after the procedure for biliary enteric anastomosis. Intraoperative photos obtained before and after the anastomosis are shown in Fig. 2(a) and (b).

### Assessment

Residual tumor staging after surgical excision was evaluated by intraoperative frozen pathological section, thus a R0 resection was defined as no tumor found in an intraoperative frozen pathological section of the reserved bile duct radical. A peritoneal drain was placed to monitor the volume, content and color of the peritoneal drainage. Postoperative complications were evaluated and staging by Clavien-Dindo classification [13]. Postoperative complications, such as anastomotic leakage, abdominal cavity bleeding, hemorrhage of the digestive tract, including hemobilia and gastrointestinal bleeding, biliary tract infection and abdominal infection, were observed. The diagnostic criteria for a bile leak were defined as bile discharge from an abdominal wound and/or drainage with a total bilirubin level of > 5 mg/mL or three times the serum level or cholangiographic evidence of dye leaking from the opacified bile ducts [14].

Liver function tests, ascites, and encephalopathy were monitored during follow-up visits to assess for liver failure. Resumption of gastrointestinal function was closely observed. Abdominal infection, abdominal abscess and gastrointestinal hemorrhage were diagnosed according to the symptoms of fever, abdominal pain, serologic indicators and ultrasonography, CT and endoscopy.

As for follow up assessment, a comprehensive examination including CT/MRI and blood test (including routine blood test, biochemical, serum tumor marker especially CEA, and CA19–9 was performed 1 month after discharge and every 3–4 months thereafter (CEA: CarcinoEmbryonic Antigen, CA19–9: Carbohydrate Antigen 19–9). Besides, abdominal pain, digestive function, etc., were closely observed.

### Statistical analysis

All statistical analyses were performed with SPSS software, version 17.0 (SPSS, Inc., Chicago, IL).

### Results

Patient demographics and clinical characteristics are shown in Table 1. From August 2013 to December 2015, 65 patients with stage III/IV HCCA were admitted to our center; of these patients, 61 underwent resection with curative intent followed by hepaticojejunostomy. Thirty-four of the 61 patients underwent resection segment 4(or plus resection segment 1) and basin-shaped hepaticojejunostomy, 2 patients were lost to follow-up, and the remaining 32 cases were enrolled in our study. No history of underlying liver disease was present.

**Table 1:** Patient demographics and characteristics

Sex		No./Median Value
Male		17
Female		15
Age (years)		59 (±10.9) y
Preoperative concurrent diseases		
	Hypertension	6
	Diabetes	5
	Cardiovascular and pulmonary disease	0
Previous history of surgery		
	Yes	6
	No	26
Preoperative blood test		
	CA19–9 (U/L)	397.25 (±54.18)
	CEA (ng/L)	4.34 (±36.91)
	ALB (g/L)	39.4 (±5.01)
	PT (s)	11.6 (±1.56)
	Total bilirubin (μmol/L)	193.15 (±159.09)

All of the patients were jaundiced on admission with a mean total bilirubin level of 193.15 mg/dL (±159.09). Most of the patients (25/32) had elevated serum CA19–9 levels. All of the patients were evaluated for whether it was feasible to perform exploratory laparotomy under general anesthesia, based on sufficient cardiopulmonary and renal function.

The surgical strategy was based on the location and involvement of the tumor mass in an effort to achieve an R0 resection. All of the patients in our study underwent resection segment 4(or plus resection segment 1); 22 patients underwent procedures combined with segment I resection. Portal venous invasion was detected macroscopically in 8 patients (25%) during surgery, while hepatic arterial invasion was detected macroscopically in 4 patients (12.5%) of the 8 which had portal venous invasion (Table 2). According to postoperative histopathological examination, 9 cases had lymph node metastasis, 19 cases had perineural infiltration and 9 had hilar fatty tissue infiltration. Concomitant vascular resection was performed due to macroscopic involvement, with partial portal vein resection in 8 patients and hepatic artery resection in 4 patients. As a consequence of resection segment 4(or plus resection segment 1) for radical resection of hilar cholangiocarcinoma, all of the patients had abundant bile stumps, ranging in number from 4 to 8 (Table 2). Basin-shaped hepaticojejunostomy was performed in every case in this study regardless of the number of bile duct stumps (Fig. 2). The median operating time for hepato-enteric anastomosis was 42 min (ranging from 25 to 58 min). Intra-operative blood loss ranged from 180 to 1200 mL. Four patients received blood transfusions (ranging from 2 to 4 units).

**Table 2:** Clinical data of operations

Vascular invasion		
	Positive/Total	Proportion
PVI	8/32	25%
HAI	4/32	12.5%
Number of biliary stumps		Number of cases
	3	3
	4	13
	5	9
	6	3
	7	3
	8	1
Intra-operative blood loss, mL		Median value (SD)
		350 (212.9)
Duration of hepato-enteric anastomosis, minutes		Median value (SD)
		42 (8.5)

*PVI* Portal Vein Invasion, *HAI* Hepatic Artery Invasion

Note: 8 cases underwent portal vein resection following reconstruction, including 4 cases that underwent artery resection simultaneously

The postoperative recovery, including time to gurgling sound, time to anal exhaust and mean duration of postoperative hospitalization, are shown in Table 3.

**Table 3:** Postoperative recovery and postoperative complications

Postoperative recovery and hospital stay		
	Recovery time of gurgling sound (H)	48.8 ± 43.2
	Recovery time of anal exhaust (H)	72.0 ± 46.9
	Postoperative hospital stay (D)	14.0 ± 5.9
Postoperative complications		No. (percentage)
Intra-abdominal hemorrhage		1 (3.1%)
	Grade I	0
	Grade II	1
Gastrointestinal hemorrhage		1 (3.1%)
	Grade I	0
	Grade II	1
Bile leakage		3 (9.3%)
	Grade I	1
	Grade II	2
Biliary infection		3 (9.3%)
	Grade I	0
	Grade II	3
Intra-abdominal infection		2 (6.2%)
	Grade I	0
	Grade II	2
Pulmonary infection/Pleural effusion		2 (6.2%)
	Grade I	0
	Grade II	1
	Grade IIIa	1
Incision infection		1 (3.1%)
	Grade I	1
	Grade II	0
Hepatic renal failure		0

Postoperative complications were evaluated and staging by Clavien-Dindo classification

There were no perioperative deaths. Postoperative complications are shown in Table 3. The most common complications were bile leakage (3/32, 9.3%) and biliary infection (3/32, 9.3%), while intra-abdominal hemorrhage, hemorrhage of the digestive tract, pulmonary infection or pleural effusion were also observed in our study. These complications prolonged hospitalization but were all managed after corresponding treatment.

**Fig. 3:**
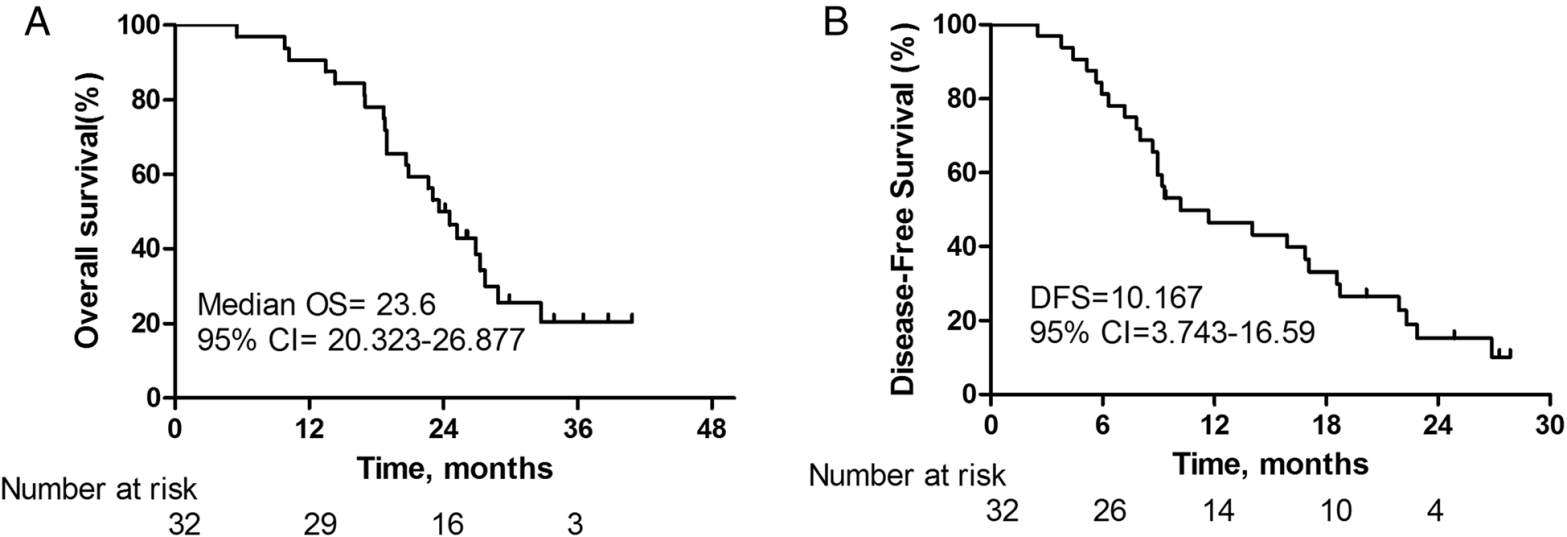
Survival data of study cohort. **a** Overall survival of patients undergo surgery with curative resection and basin-shaped hepaticojejunostomy. **b** Disease-free survival of patients undergo surgery with curative resection and basin-shaped hepaticojejunostomy. 95% CI, 95% confidence interval

All of the patients were histologically diagnosed with advanced cholangiocarcinoma, positive lymph node invasion was found in 12 patients, and perineural invasion was found in 15 patients; in addition, capsular infiltration was found in 7 patients. According to frozen sections, as well as the definitive histological examination, curative resection was performed in all of the patients. At the endpoint of follow-up, 14 patients died from tumor recurrence and progression. In these 14 patients, 3 were found pulmonary metastasis, 5 were found hilar lymph node metastasis, 9 were found liver metastases (combined with lymph node metastasis), 2 were found peritoneal implantation metastasis. However, none of these patients had evidence to demonstrate local recurrence around the hepato-enteric anastomosis. The median follow up time was 26.167(95% CI = 21.917–30.416) months and the mean overall survival time was 23.6(95% CI = 20.323–26.877) months in this study (Fig. 3).

## Discussion

The main purpose of this study was to introduce and practice basin-shaped hepaticojejunostomy, followed by segment IV/extended IV and high hilar resection of stage IV hilar cholangiocarcinoma, in which the tumor invaded the bilateral secondary (tertiary) bile ducts. The outcomes data revealed that the basin-shaped hepaticojejunostomy was safe and effective for patients with stage III/IV hilar cholangiocarcinoma after resection segment 4(or plus resection segment 1) and high hilar resection.

Hilar cholangiocarcinoma is the most common malignant tumor affecting the extrahepatic bile duct [15], constituting 46–97% of all bile duct cancers [16, 17]. Due to the relatively slow growth and concealed location of the tumor, most patients do not present with any symptoms until the late stage, with unresectable tumors. Therefore, most patients have a poor prognosis, except for very few patients with indolent and slow-growing hilar cholangiocarcinoma, who can attain long-term survival [18]. While palliative procedures, such as photodynamic therapy (PDT) and transarterial chemoembolization, have been reported to prolong survival [19, 20], the only potentially curative therapy for hilar cholangiocarcinoma is R0 resection [21]. Development of a more aggressive approach to HCCA, involving radical biliary resection with segmental hepatic resection inclusive of the caudate lobe, [22–25] resulted in dramatic improvements in survival and decreases in local tumor recurrence. However, the postoperative morbidity and complication rates remained high [26, 27]. This contradiction was caused by the asynchrony of improved resection techniques with relatively underdeveloped reconstruction techniques.

Because the main goal of surgical resection is to achieve adequate resection margins,[24] stage III/IV HCCA usually requires left- or right-sided hepatectomy, and it always requires caudate lobe resection [28]. Such resections can provide a curative prognosis, but they are associated with increased difficulty of biliary reconstruction because the resection leaves more biliary stumps. The management of these remaining biliary stumps can greatly affect surgical outcomes and patient recovery.

In contrast, the common postoperative complications of HCCA include intra-abdominal hemorrhage, hemorrhage of the digestive tract, bile duct leaks and biliary infection. Most of these complications are related to the hepato-enteric anastomosis [29].

Therefore, the exploration of new methods to improve the postoperative complication rate is worthwhile. Thus, we assessed whether an improved hepaticojejunostomy might simplify the procedure, shorten the operation time and decrease the postoperative complication rate.

With the conventional method, biliary continuity is restored with a Roux-en-Y of jejunum loop, and the hepato-enteric anastomosis is performed in an end-to-side manner one by one [28]. This method is effective for 1–3 stumps; for more than 4 stumps, this one-by-one anastomosis approach is time consuming, laborious and prone to errors and omissions. Although, in most hilar resection operations, the biliary stumps numbered fewer than 4, for patients with stage III/IV HCCA who underwent resection segment 4(or plus resection segment 1), the biliary stumps always numbered more than 4, and the conventional method was never helpful.

In response to this situation, we practiced the basin-shaped anastomosis method, which sutures the jejunum and the sheath of the Glisson pedicle around the biliary stumps to shape a basin, after which the biliary stumps are inserted into the basin-shaped anastomosis, avoiding the one-by-one procedure.

The Glisson capsule extends into the liver as tissue sheaths around the hepatic ducts, hepatic arteries, and portal tributaries [30]. Primary, secondary and tertiary biliary ducts are surrounded by the Glisson’s capsule completely and are connected to each other in common tunnels. Thus, suturing of the peripheral Glisson’s capsule surrounding the biliary duct will include all of the passing biliary duct in the common tunnel and will shape the “basin” for our biliary-enteric anastomosis technique. The methods of suturing the ligament surrouding the biliary ducts for hepaticojejunostomy have been introduced and practiced in different studies, and the safety and efficacy have been demonstrated and verified [31–33]. Similar to but different from these methods, we made some improvements with the aim of reducing the postoperative complication rate: 1. Each biliary stump was inserted a polyvinyl chloride tube as a stent. The secondary and tertiary biliary stumps were always tiny and easy to constrict by inflammatory edema, and the inserted stent could help to keep the tunnels open and unobstructed. The stent will come loose and fall off after regression of the local inflammation and will finally be excreted through the intestine. The lost stent is not new technique, but was rare seen in research about hepatic-portal to jejunum anastomosis. We tried to apply this technique to our hepaticojejunostomy to observe and discuss if it could be an improvement for basin-shaped hepaticojejunostomy. In our study, 17 cases were applied the stent while 3 cases had 3 biliary radicals, 12 cases had 4 biliary radicals and 2 cases had 5 biliary radicals. The decision was made according to the lumen size of biliary radicals, a stent was inserted if the lumen of biliary radical was not too tight nor too loose. According to our standard, the use of stent were mostly occurred in cases with less number but larger lumen size of biliary radicals, the internal control group had critically selective bias to the stent group. As the results had approved, all complications of bile leakage and biliary infection were found in the no-stent group, as well as hemorrhage. 2. Different from previous methods that suture the peripheral Glisson’s capsule to surround the biliary stumps, our method sutures the biliary wall together with the peripheral Glisson’s capsule to form the wall of the anastomosis, except for some parts for which there were no biliary stumps along the suture path. This method could fix the biliary stumps to prevent motion, and it works together with the inserted stent to keep the tunnels open. Compared with previous studies [5, 6] associated with stage III/IV HCCA, our improved method demonstrated advantages of a lower complication rate and shorter operation time. The reasons could be the following: 1. When multiple bile duct stumps were addressed with the basin-shaped method, all of the stumps were inserted into one common tunnel; the single anastomosis not only saved time but also allowed for sufficient space to ensure exact and complete suturing. 2. Furthermore, the bile ducts were anastomosed in their original locations, this avoiding the retraction and pulling that can increase the suture tension and the risk of laceration. With the conventional method of suturing the anastomosis one by one, the completed anastomosis will affect or even occupy the space for the next anastomosis, rendering the operation more difficult and uncertain. 3. Moreover, multiple anastomoses cannot ensure avoidance of  tension for every anastomosis; later sutures can retract previous ones to increase the tension, which can cause tearing of the anastomosis.

The limitations of this study were defined by its small sample size and lack of randomized controls. Further prospective studies, with large sample sizes and randomized controls, are needed to demonstrate the effectiveness. Furthermore, with this surgical technique for tumor disease, the risk of tumor recurrence remains unknown for its short follow-up observation period. Prolonged follow-up durations for the observation of tumor recurrence are needed to demonstrate oncologic safety.

## Conclusions

Basin-shaped biliary-enteric anastomosis for patients with stage III/IV HCCA following IV/extended IV segment resection was safe and effective. Advantages in postoperative complications and operation time could be present with this method.

## Data Availability

The datasets used and/or analyzed during the current study available from the corresponding author on reasonable request.

## Abbreviations

CA19–9: Carbohydrate Antigen 19–9
CEA: CarcinoEmbryonic Antigen
CT: Computed X-ray Tomography
HCCA: Hilar cholangiocarcinoma
MRCP: Magnetic Resonance CholangioPancreatography
NCCN: National Comprehensive Cancer Network
TBil: Total Bilirubin
